# Innate immune cell barrier-related genes inform precision prognosis in pancreatic cancer

**DOI:** 10.3389/fimmu.2025.1559373

**Published:** 2025-05-23

**Authors:** Qiang Luo, Tingting Jiang, Dacheng Xie, Xiaojia Li, Keping Xie

**Affiliations:** ^1^ Center for Pancreatic Cancer Research, The South China University of Technology School of Medicine, Guangzhou, Guangdong, China; ^2^ Center for Pancreatic Cancer Center, The South China University of Technology Comprehensive Cancer Center, Guangzhou, Guangdong, China; ^3^ Department of Medical Oncology, Shanghai Pulmonary Hospital & Thoracic Cancer Institute, Tongji University School of Medicine, Shanghai, China

**Keywords:** ensemble machine learning, prognostic model, pancreatic cancer, innate, immunology, UBASH3B

## Abstract

**Introduction:**

Pancreatic cancer (PC) remains a lethal malignancy with limited treatment options. The role of innate immune cell barrier-related genes in PC prognosis is poorly defined. This study aimed to identify prognostic biomarkers, develop a predictive model, and uncover novel targets for personalized therapy.

**Methods:**

Innate immune cell barrier-related genes were curated from KEGG, ImmPort, MSigDB, and InnateDB. Differential expression analysis was performed using TCGA and GTEx datasets. Univariate Cox regression identified survival-associated genes. Prognostic modeling of PC was developed using 14 machine learning algorithms, with performance validated through long-term survival metrics, functional enrichment, immune infiltration analysis, and drug sensitivity profiling. Core genes were prioritized via the "mime1" package, and single-cell RNA sequencing (scRNA-seq) data explored UBASH3B’s functional role.

**Results:**

352 differentially expressed genes of Innate immune cell barrier-related were identified, with NK cell pathways linked to PC immunity. Univariate Cox analysis revealed 8 protective and 84 risk genes. The RSF model (trained on risk genes) showed strong 3- and 5-year survival prediction. High-risk patients exhibited elevated tumor mutation burden (TMB), reduced NK/CD8+ T cell infiltration, and resistance to Erlotinib/Oxaliplatin but sensitivity to 5-Fluorouracil. Five key genes (ITGB6, COL17A1, MMP28, DIAPH3, UBASH3B) were highlighted. UBASH3B, a novel marker, correlated negatively with NK cell activation and mediated immune signaling and drug resistance.

**Discussion:**

This study established the CDRG-RSF model, a robust prognostic tool leveraging innate immune genes. UBASH3B’s dual role in immune suppression and drug resistance highlights its potential for stratifying PC patients into tailored treatment groups. The findings underscore the importance of integrating machine learning with immune profiling to advance precision oncology for PC.

## Introduction

Pancreatic cancer (PC) is an exceptionally aggressive malignancy with a rising incidence globally and an extremely low five-year survival rate of approximately 10% (1, 2). This dismal prognosis primarily stems from the lack of early symptoms, leading to most patients being diagnosed at advanced stages when optimal treatment opportunities have passed. Additionally, PC exhibits high resistance to existing therapies such as surgery, chemotherapy, and radiotherapy, further complicating treatment efficacy. The significant inter-patient variability in response to these treatments adds another layer of complexity to therapeutic decision-making (3). To improve patient outcomes, there is an urgent need to identify novel biomarkers that can guide personalized treatment strategies for PC.

The innate immune system serves as the body’s first line of defense and plays a dual role in cancer: it can suppress tumor development through immune surveillance mechanisms but may also promote tumor progression via chronic inflammation (4). In the PC microenvironment, the innate immune cell barrier predominates (5). Specifically, monocytes/macrophages, dendritic cells, natural killer (NK) cells, and neutrophils form a complex barrier influencing tumor development and metastasis (6). NK cells, in particular, play a crucial role in immune surveillance and control of PC. Their ability to recognize and rapidly respond to abnormal cells without prior sensitization endows them with potent anti-tumor capabilities. In PC, patient survival rates correlate positively with the relative frequency of NK cells in their blood, which exhibit lower cytotoxicity compared to healthy individuals (7). Despite numerous clinical trials targeting the innate immune system in PC, most efforts have not yielded significant improvements (8, 9), underscoring the need for more suitable targets to enhance immunotherapy strategies, especially those involving NK cell inhibitors.

UBASH3B (Ubiquitin Associated and SH3 Domain Containing B), also known as T-cell ubiquitin ligase 1 (TULA-1), is a protein involved in immune regulation. It participates in intracellular signaling pathways, particularly those associated with SH3 binding domains and ubiquitin ligase activity. Research indicates that UBASH3B can modulate T-cell receptor signaling, thereby inhibiting T-cell activation (10). In cancer studies, UBASH3B has been implicated in promoting tumorigenesis across various types of cancers, including prostate cancer (11), breast cancer (12), and leukemia (13). However, its role in PC remains largely unexplored.

Advances in machine learning technology have opened new avenues for biomedical research. Machine learning algorithms can process high-dimensional data and uncover hidden patterns, making them particularly effective for identifying valuable biomarkers from large gene expression datasets (14). Therefore, this study leverages ensemble machine learning techniques to explore whether genes related to the innate immune cell barrier can serve as potential prognostic markers for PC. We aim to construct an effective prognostic risk model and identify key genes. By integrating the latest bioinformatics tools and clinical data, our research seeks to provide new theoretical foundations and technical support for precision medicine in PC.

## Materials and methods

### Data collection and preprocessing

We compiled a comprehensive list of genes associated with innate immune cell barriers, including monocytes/macrophages, dendritic cells, NK cells, and neutrophils, from four databases: KEGG, ImmPort Portal, MSigDB, and InnateDB. After deduplication, this process yielded a final list of 1,356 unique genes.

We integrated datasets from The Cancer Genome Atlas (TCGA) and the Genotype-Tissue Expression (GTEx) project, encompassing 178 PC samples and 172 normal pancreatic tissue samples. For the validation of our machine learning models, we subsequently collected three PC cohorts (GSE62452, GSE78229, and GSE85916) from the Gene Expression Omnibus (GEO) database (**Supplementary Table S1**). Batch effects in gene expression data were corrected using the “removeBatchEffect” function from the “limma” package to eliminate technical biases across datasets (15) (**Supplementary Figure S1**, **Supplementary Table S2**).

We obtained scRNA-seq data of 61 patients with pancreatic cancer (PC) from six GEO databases (GSE154778, GSE155698, GSE197117, GSE212966, GSE231535, GSE242230). Raw sequencing data were processed using the Seurat package (v4.3) (16). Initial quality control filtered cells with low complexity (nFeature_RNA > 200), high mitochondrial gene content (percent.mt < 20), elevated hemoglobin expression (percent.HB < 10), or extreme ribosomal gene expression (percent.Ribo < 50). To address technical variation, we selected 3,000 highly variable genes (variance-stabilizing transformation), scaled the data, and performed PCA (npcs=50) with PC quantity optimized by the Elbow method (>90% cumulative variance; **Supplementary Figure S2A**). We used the “harmony” R package to correct batch effects and integrate multiple samples, harmonizing data across batches while preserving biological variation (17) (**Supplementary Figure S2B**). Harmony-corrected embeddings were then visualized in two dimensions using UMAP (RunUMAP), and utilizing the first 50 Harmony dimensions for consistency. Cell clusters were resolved using Leiden algorithm-based graph clustering (resolution=0.5, silhouette-optimized) on Harmony-derived SNN graphs. Annotation: Cell types were annotated using the CellMarker database and standard cell-type markers for accurate identification and classification.

### Differential expression analysis

For the differential expression analysis, we utilized the “DESeq2” package (18) to identify genes that are significantly differentially expressed between PC samples and normal samples. |log2FC| > 2 and *p* < 0.001.

### Functional enrichment analysis

To interpret the biological significance of the DEGs, we conducted Gene Ontology (GO) term, Kyoto Encyclopedia of Genes and Genomes (KEGG) pathway, and Gene Set Enrichment Analysis (GSEA) enrichment analyses using the “clusterProfiler” package (19).

### Univariate and multivariate Cox regression analyses

We evaluated the prognostic significance of gene sets and clinical information in PC using univariate and multivariate Cox regression analyses with the “survival” package. Univariate analysis assessed the impact of individual factors on patient survival, while multivariate analysis identified independent predictors by adjusting for confounding variables.

### Construction of machine learning prediction models

To develop robust prognostic models for PC, we utilized 14 machine learning algorithms: Lasso, RSF, Enet, StepCox, CoxBoost, plsRcox, superpc, gbm, survival-SVM, Ridge, obliqueRSF, xgboost, CForest, and CTree, constructing a total of 162 models (**Supplementary Table S3**).

Key prognostic genes were first identified using RSF, Lasso, StepCox, and CoxBoost. These genes were then used to build models with all 14 algorithms. Model performance was ranked by the average c-index from three validation sets and further evaluated using univariate and multivariate Cox regression, Kaplan-Meier analysis, and ROC curve AUC.

Riskscore cutoffs were systematically defined through a three-phase process: First, in the TCGA-PAAD training cohort, the median risk score was selected as the primary threshold to balance group sizes (High_risk: n = 87 vs. Low_risk: n = 87). Second, external validation cohorts underwent removeBatchEffect batch correction followed by cutoff application: the TCGA median was retained unless cohort-specific median deviated beyond ±5%, in which case adjusted thresholds were used.

### Tumor Mutation Burden, ESTIMATE, and CIBERSORT Analysis

To characterize the genomic and immune profiles of pancreatic adenocarcinoma (PAAD) from TCGA: 1. TMB Analysis: We obtained single-nucleotide variation data using the “TCGAbiolinks” package (20) and analyzed mutation burdens with “Maftools” package (21). 2. ESTIMATE Analysis: Using RNA-seq data, we calculated ImmuneScore, StromalScore, and TumorPurity scores with the “ESTIMATE” package (22) to assess immune and stromal infiltration. 3. CIBERSORT Analysis: We performed deconvolution using the “CIBERSORT” package (23) to estimate the abundance of various immune cell subpopulations.

### Drug sensitivity analysis

To evaluate drug sensitivity across different subtypes, we obtained the GDSC2 expression and resistance database files (GDSC2 Expr.rds and GDSC2 Res.rds) from the Genomics of Drug Sensitivity in Cancer (GDSC) website (24). Using the “oncoPredict” package (25), we assessed the sensitivity of various drugs for each subtype identified in our study.

### Identification of key genes

To identify the most important genes in our prognostic model, we employed ensemble machine learning methods using the “mime” package (26). Specifically, we constructed a total of 18 models utilizing eight survival analysis algorithms: Lasso, Enet, Boruta, CoxBoost, RSF, XGBoost, StepCox, and SVM-REF. Core features were determined by ranking genes based on their frequency of selection across these models.

The top five genes identified as most significant were further analyzed for differential expression between normal and tumor tissues using the GEPIA database. Additionally, we evaluated these genes in relation to tumor stage and survival outcomes in PC patients.

## Results

### Screening and enrichment analysis of differentially expressed genes related to the innate immune cell barrier

To investigate whether genes associated with the innate immune cell barrier could serve as potential markers for predicting prognosis in PC, we compiled a comprehensive list of such genes from multiple databases including KEGG, ImmPort Portal, MSigDB, and InnateDB. After removing duplicates, this compilation resulted in a set of 1,356 unique genes related to monocytes/macrophages, dendritic cells, NK cells, and neutrophils (**Supplementary Table S4**).

**Figure 1:**
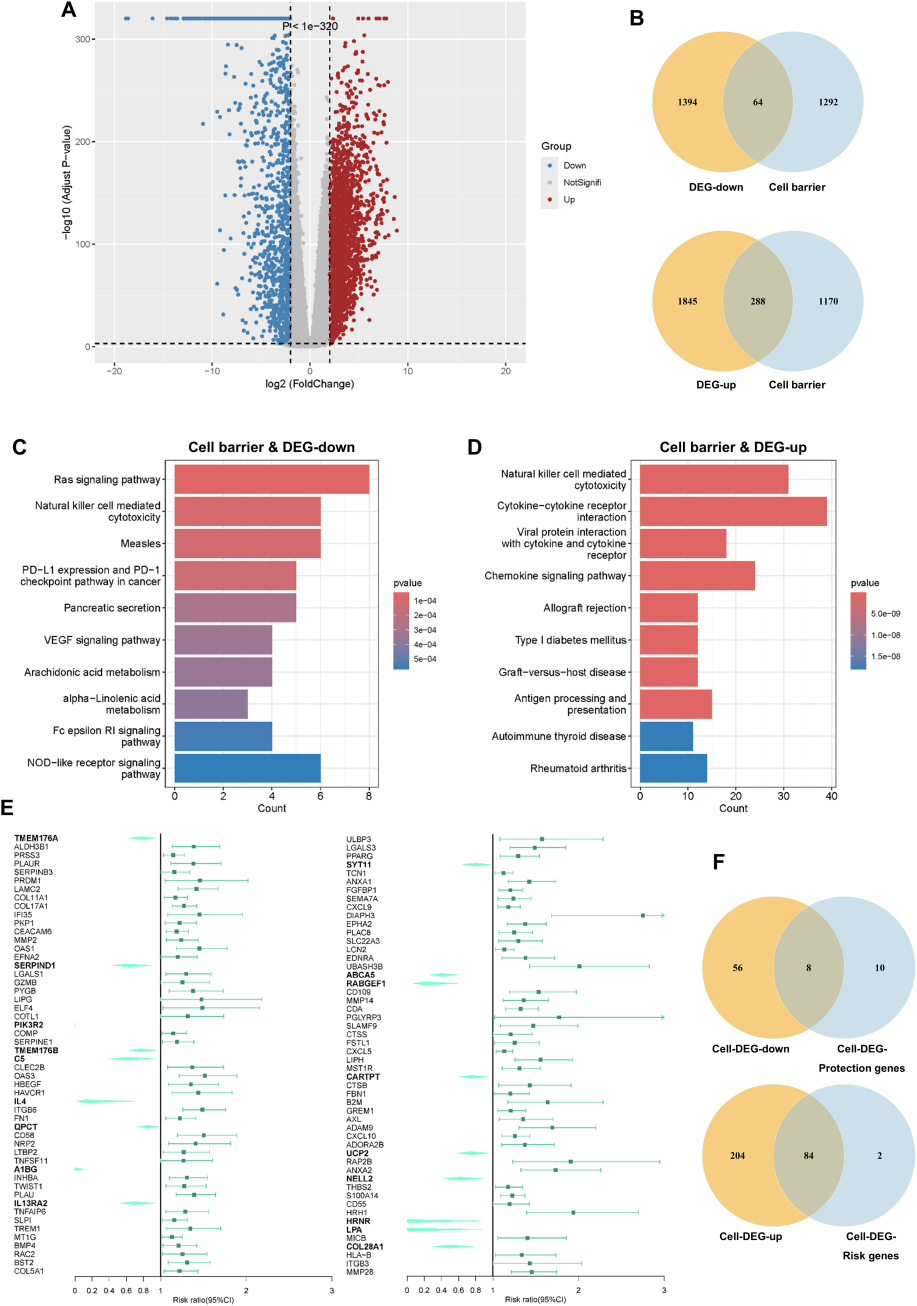
Screening and enrichment analysis of gene sets. **(A)** Volcano plot depicting DEGs between normal and pancreatic cancer tissues. **(B)** Venn diagram illustrating the overlap between innate immune barrier-related genes and DEGs. **(C, D)** Top ten KEGG pathways enriched in Cell-DEG-down and Cell-DEG-up genes. **(E)** Forest plot from univariate Cox regression analysis for all genes in Cell-DEG-down and Cell-DEG-up. **(F)** Venn diagrams showing the intersection of Cell-DEG-down and Cell-DEG-up with protection genes and risk genes.

We integrated data from TCGA and the GTEx project, encompassing 178 PC samples and 172 normal pancreatic tissue samples. Using the “DESeq2” package for differential expression analysis, we identified 3,591 DEGs, comprising 1,458 downregulated (DEG-down) and 2,133 upregulated (DEG-up) genes (**Figure 1A**). By intersecting these DEGs with our curated list of innate immune cell barrier-related genes, we refined our focus to genes specifically relevant to this study (**Figure 1B**).

Subsequent functional enrichment analyses using GO and KEGG pathways revealed significant associations. GO enrichment indicated that both Cell Barrier & DEG-down (Cell-DEG-down) and Cell Barrier & DEG-up (Cell-DEG-up) genes were prominently linked to leukocyte-mediated immune responses, regulation of immune effector processes, and myeloid leukocyte-specific functions (**Supplementary Figures S3A, B**). KEGG pathway analysis highlighted the involvement of the innate immune cell barrier in critical aspects of PC development, including immune escape, metabolic reprogramming, and abnormal signal transduction. Notably, pathways such as NK cell-mediated cytotoxicity and chemokine signaling underscored the pivotal role of NK cells in the innate immune barrier of PC (**Figures 1C, D**).

To identify genes with prognostic significance, we performed univariate Cox regression analysis on the 352 overlapping genes. This analysis identified 104 prognosis-associated genes (CDGs), comprising 18 protective genes and 86 risk genes (**Figure 1E**). Further cross-referencing with the Cell-DEG-down or Cell-DEG-up confirmed 8 protective genes (CDPGs) and 84 risk genes (CDRGs) (**Figure 1F**; **Supplementary Table S4**), providing a robust foundation for constructing subsequent prognostic models.

### Development of a prognostic risk model for PC based on the innate immune cell barrier using machine learning integration

**Figure 2:**
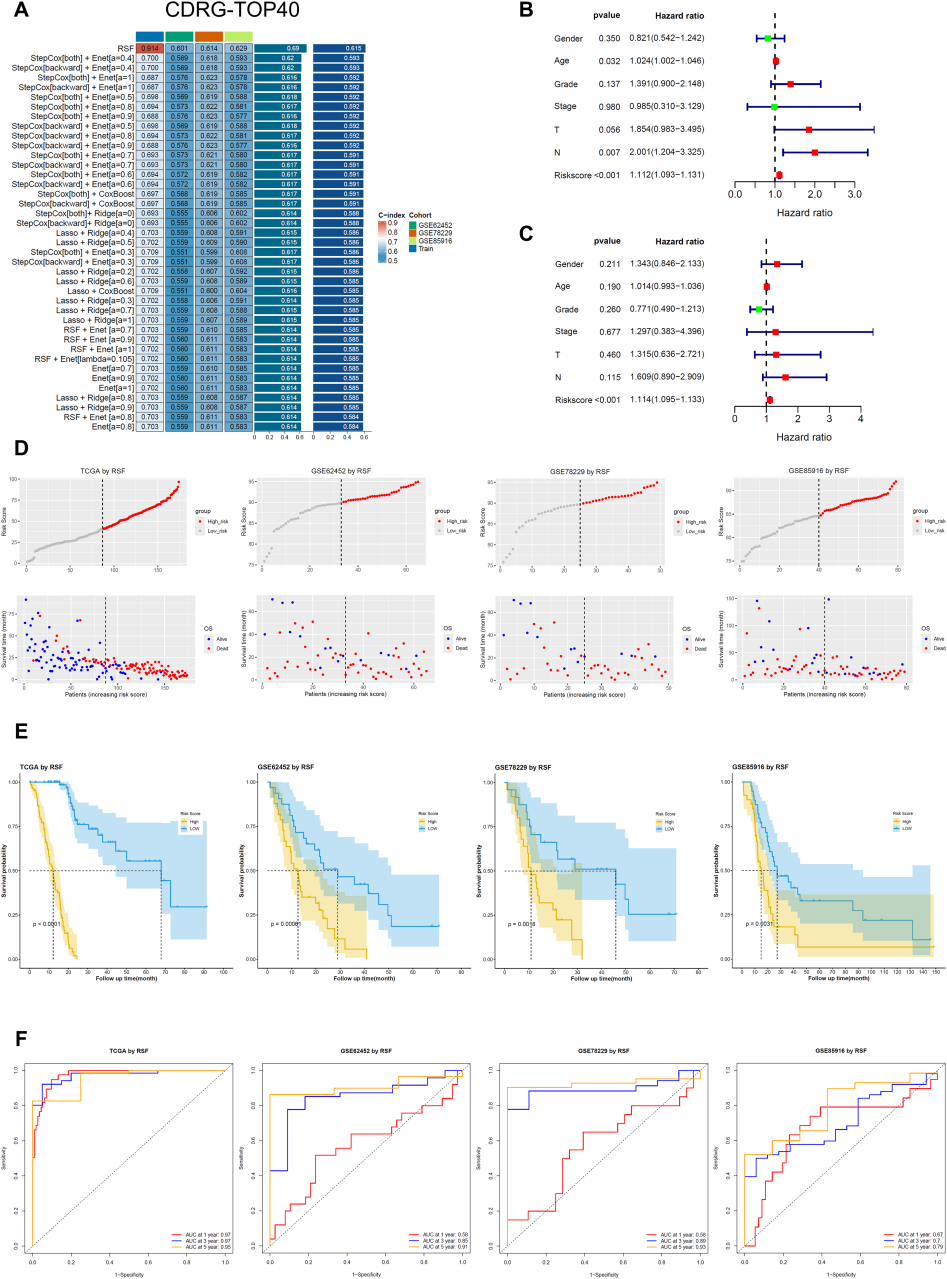
Construction and validation of machine learning models based on CDRGs. **(A)** Top 40 machine learning models ranked by average C-index, constructed using CDRGs as features among 162 models. **(B, C)** Univariate and multivariate Cox regression analysis of RiskScore predicted by the Random Survival Forest (RSF) model, based on clinical information from the TCGA-PAAD cohort. **(D)** Scatter plots showing RiskScores predicted by the RSF model in the training set (TCGA-PAAD) and three validation sets (GSE62452, GSE78229, and GSE85916). **(E)** Kaplan-Meier (KM) survival curves for the training set and validation sets based on RSF-predicted RiskScores. **(F)** Time-dependent ROC curves for predicting 1-year, 3-year, and 5-year overall survival (OS) in the training set and validation sets.

To develop a robust prognostic risk model based on the innate immune cell barrier for PC, we selected CDGs, CDPGs, and CDRGs as input features. We fitted 162 prediction models using the TCGA-PAAD dataset and validated these models with three independent cohorts: GSE62452, GSE78229, and GSE85916.Model performance was evaluated by calculating the concordance index (c-index) in each dataset. The results indicated that the RSF model, based on the CDRG gene set, performed the best, achieving an average c-index of 0.615 across all validation datasets (**Figure 2A**; **Supplementary Table S5**).

To further assess the clinical utility of our model, we conducted univariate and multivariate Cox regression analyses incorporating clinical information from the TCGA cohort with the generated Riskscore. The results confirmed that Riskscore served as a significant independent prognostic factor (HR=1.114, 95%CI 1.095-1.133, *p* < 0.001) (**Figures 2B, C**). Patients were stratified into high-risk and low-risk groups based on the median Riskscore value. As shown in **Figure 2D**, there were significant differences in Riskscore distribution, survival status, and time between the two groups.

Kaplan-Meier survival analysis demonstrated that patients in the low-risk group exhibited significantly better overall survival across all four cohorts (*p* < 0.01) (**Figure 2E**). Additionally, we evaluated the predictive performance of the model using receiver operating characteristic (ROC) curves. **Figure 2F** illustrates the accuracy of the model in predicting 1-year, 3-year, and 5-year survival rates across the four cohorts. While the AUC values for 1-year survival were lower in the three validation cohorts, the AUC values for 3-year and 5-year survival exceeded 0.7 in all four cohorts, with AUC values close to or exceeding 0.9 in three of the four cohorts (excluding GSE85916). This indicates that our model exhibits excellent long-term predictive performance.

### Function enrichment and tumor mutation burden analysis based on risk score

**Figure 3:**
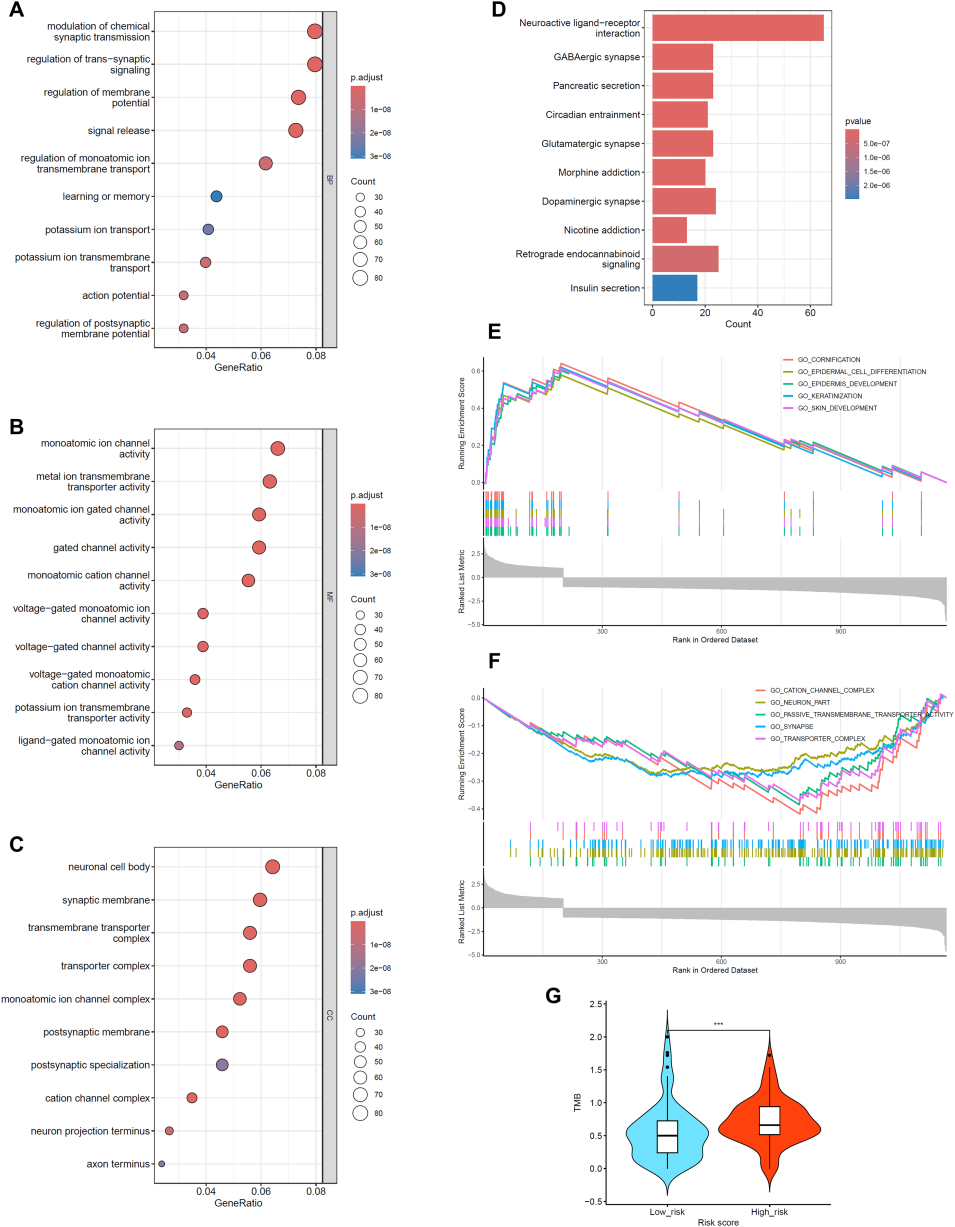
Functional enrichment and TMB Analysis in high- and low-risk populations. Based on TCGA-PAAD cohort data. **(A-C)** Gene Ontology (GO) enrichment analysis for Biological Process (BP), Molecular Function (MF), and Cellular Component (CC) categories, showing only the top 10 pathways. **(D)** KEGG pathway enrichment analysis, displaying only the top 10 pathways. **(E, F)** Gene Set Enrichment Analysis (GSEA) results, showing the top 5 positively regulated and top 5 negatively regulated pathways. **(G)** Violin plot illustrating Tumor Mutational Burden (TMB) distribution between high-risk and low-risk groups, ***p < 0.001 compared to Low_risk group.

To elucidate the functional differences between high-risk and low-risk groups, we stratified the TCGA-PAAD cohort based on the median Riskscore value into high-risk and low-risk groups. We then compared DEGs between these two groups and performed comprehensive functional enrichment analyses. GO analysis revealed that molecular function differences were primarily focused on ion balance, metabolic regulation, neural signal transmission, and intercellular communication (**Figures 3A–C**). KEGG pathway analysis further validated significant differences in neurological functions, affecting neural signal transmission, pain management, reward mechanisms, and metabolic processes, all of which are directly associated with pancreatic function and disease status (**Figure 3D**).

GSEA highlighted biological process differences between the two groups: the high-risk group was closely associated with epithelial tissue development and differentiation processes, including epithelial cell differentiation, keratinization, and keratinocyte formation; whereas, the low-risk group exhibited a greater focus on neuronal and synaptic functions, ion channel activity, and substance transport processes (**Figures 3E, F**). These findings suggest distinct cellular and tissue-level functional differences among different risk groups.

Additionally, the high-risk group showed significantly higher TMB compared to the low-risk group (*p* < 0.001) (**Figure 3G**), indicating more complex genomic instability in the high-risk group.

### Immune infiltration analysis and drug sensitivity prediction based on risk score

**Figure 4:**
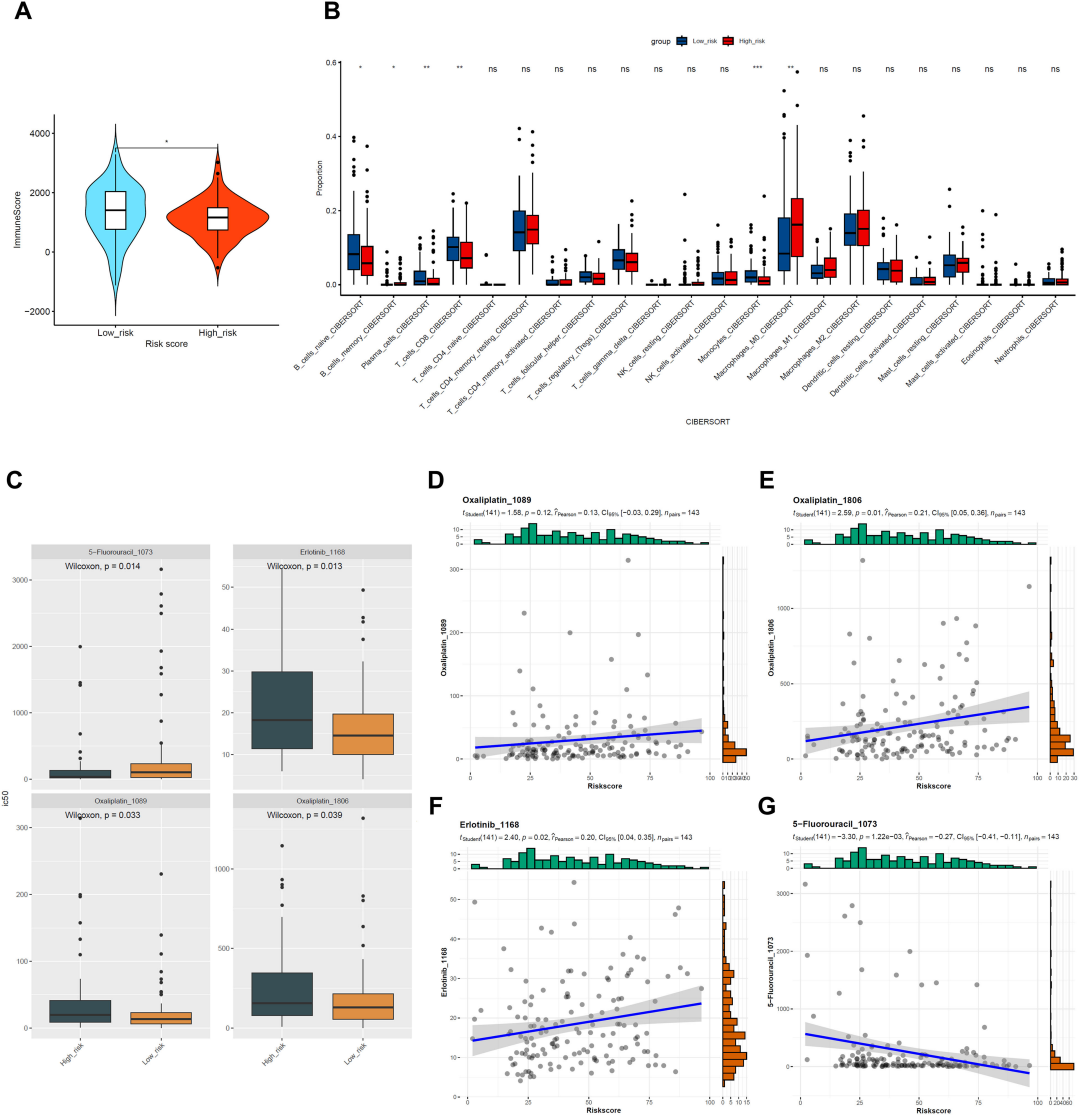
Differences in immune infiltration and drug sensitivity between high- and low-risk groups. Based on TCGA-PAAD cohort data. **(A)** Violin plot showing the distribution of ImmuneScore from ESTIMATE analysis between high- and low-risk groups, *p < 0.05 compared to Low_risk group. **(B)** Box plots displaying the results of CIBERSORT analysis for immune cell infiltration in high- and low-risk groups; *p < 0.05, **p < 0.01, ***p < 0.001 compared to Low_risk group. **(C)** Box plots comparing the predicted IC50 values for 5-Fluorouracil-1073, Erlotinib-1168, Oxaliplatin-1089, and Oxaliplatin-1806 between high- and low-risk groups. **(D–G)** Scatter plots illustrating the correlation between RiskScore and the predicted IC50 values for 5-Fluorouracil-1073, Erlotinib-1168, Oxaliplatin-1089, and Oxaliplatin-1806.

To investigate the relationship between Riskscore and immune infiltration, we analyzed the TCGA-PAAD cohort using the “ESTIMATE” package. Results indicated lower immune scores (*p* < 0.05) and stromal scores (*p* > 0.05) in the high-risk group, corresponding to increased tumor purity (*p* > 0.05) (**Figure 4A**; **Supplementary Figures S4A, B**). This suggests reduced immune infiltration in the high-risk group. Furthermore, analysis of immunotherapy response using the TIDE algorithm revealed no statistically significant differences between high- and low-risk groups in TIDE scores, immune exclusion, dysfunction, or microsatellite instability (MSI) (all *p* > 0.05; **Supplementary Figures S4C-F**).

Further analysis using the “CIBERSORT” package revealed that, except for M0 macrophages and memory B-cells, the relative abundance of naive B-cells, plasma cells, CD8 T-cells, and monocytes was significantly lower in the high-risk group compared to the low-risk group (**Figure 4B**). This indicates weakened immune infiltration in the high-risk group, potentially impacting patient prognosis.

Drug sensitivity differences between the high-risk and low-risk groups were evaluated using the “oncoPredict” package. Among 198 predicted drugs, 100 showed significant differences between the two groups (**Supplementary Table S6**). Notably, IC50 values for Erlotinib, Oxaliplatin, and 5-Fluorouracil varied significantly. Specifically, IC50 values for Erlotinib and Oxaliplatin were higher in the high-risk group (*p* < 0.05), while those for 5-Fluorouracil were lower (*p* < 0.05) (**Figure 4C**). Correlation analysis confirmed positive correlations between Riskscore and IC50 values for Erlotinib and Oxaliplatin, and a negative correlation with 5-Fluorouracil (*p* < 0.05) (**Figures 4D–G**). Although Riskscore correlated positively with Oxaliplatin’s IC50, this result was not statistically significant (*p* > 0.05). Overall, the high-risk group exhibited higher drug resistance but greater sensitivity to 5-Fluorouracil, suggesting prioritizing its use in clinical treatment plans for high-risk patients.

### Key gene screening in the prognostic risk model

**Figure 5:**
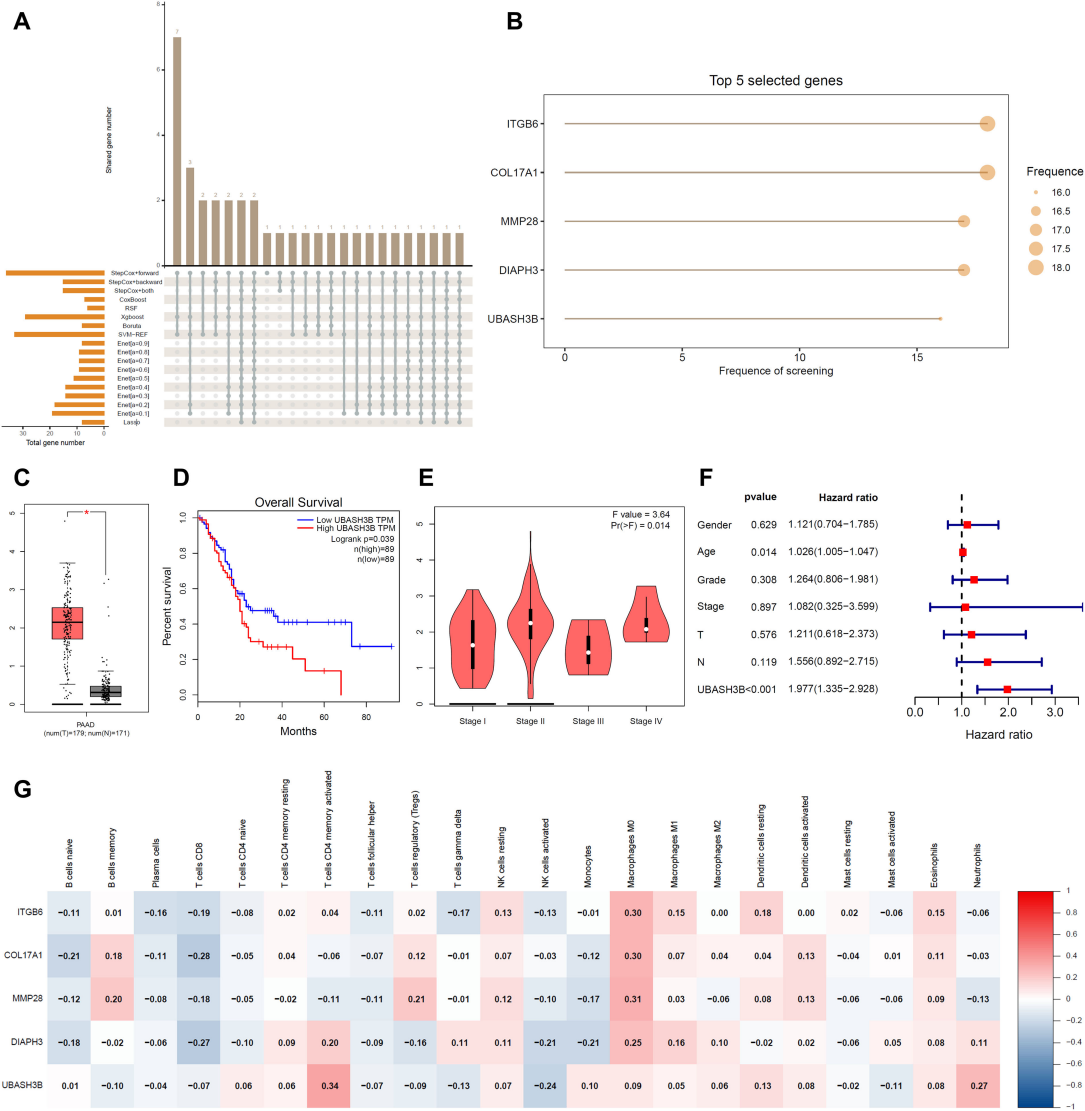
Identification of prognostic key genes. **(A)** UpSet plot showing the overlap of genes filtered by different methods. **(B)** Top 5 prognostic genes selected by 18 machine learning models for pancreatic cancer. **(C)** Box plot from the GEPIA database depicting UBASH3B gene expression levels. **(D)** Kaplan-Meier (KM) survival curve for UBASH3B expression from the GEPIA database. **(E)** Violin plot illustrating stage-wise analysis of UBASH3B expression from the GEPIA database. **(F)** Forest plot from multivariate Cox regression analysis based on UBASH3B expression data and clinical information from the TCGA-PAAD cohort. **(G)** Heatmap showing the correlation between ITGB6, COL17A1, MMP28, DIAPH3, and UBASH3B with CIBERSORT analysis results.

The RSF model with the best performance did not undergo feature screening but instead utilized all 84 genes from the CDRF set to directly construct the prediction model (**Figure 2A**). To pinpoint the most significant genes within this model, we applied the “mime” package, which utilized 18 algorithms based on 8 machine learning models (**Figure 5A**). Based on gene selection frequency, five key genes emerged as the most recurrent: ITGB6, COL17A1, MMP28, DIAPH3, and UBASH3B, each selected more than 16 times (**Figure 5B**).

We evaluated the role of these five genes in PC using the GEPIA database and multivariate Cox regression analysis (**Figures 5C–F**; **Supplementary Figure S5**). Our findings indicated that all five genes exhibited significantly higher expression levels in PC tissues compared to normal tissues (*p* < 0.05). Kaplan-Meier survival curve analysis further demonstrated a strong association between the expression of these genes and patient overall survival rates (*p* < 0.05). Multivariate Cox regression confirmed that these genes are independent prognostic factors with a significant impact on patient survival, unaffected by other variables (*p* < 0.001).

Stage staging analysis revealed that the expression levels of ITGB6, COL17A1, and UBASH3B were significantly related to the stage of PC (*p* < 0.05), suggesting their involvement in disease progression. In contrast, MMP28 and DIAPH3 did not show significant correlations with specific stages of PC development (*p* > 0.05). Given that the intersection of innate immune cell barrier-related genes and differentially expressed genes in PC primarily focuses on NK cell cytotoxicity regulation pathways (**Figures 1C, D**), we speculate that NK cell cytotoxicity plays a crucial role in PC’s occurrence and development.

To further explore the relationship between these five genes and immune infiltration, we analyzed their correlation with 22 immune cell subpopulations using the “CIBERSORT” package, with particular emphasis on NK cell subpopulations (**Figure 5G**). Notably, the expression of UBASH3B demonstrates a significant negative correlation with NK cell activation, with a correlation coefficient of approximately -0.24. Among the five identified genes, UBASH3B stands out as a potential novel prognostic marker for PC. Despite its importance, the role of UBASH3B in PC has not been previously reported in the literature (27–30), making it a focal point for future research and highlighting its potential significance in understanding and predicting PC prognosis.

### Differential expression and enrichment analysis of the UBASH3B gene

To further explore the potential mechanisms by which UBASH3B acts as a poor prognostic factor in PC, this study utilized RNA-seq data from the TCGA-PAAD database. Patients were divided into high-expression and low-expression groups based on the median UBASH3B expression level, and differential expression analysis along with functional enrichment analysis were conducted.

**Figure 6:**
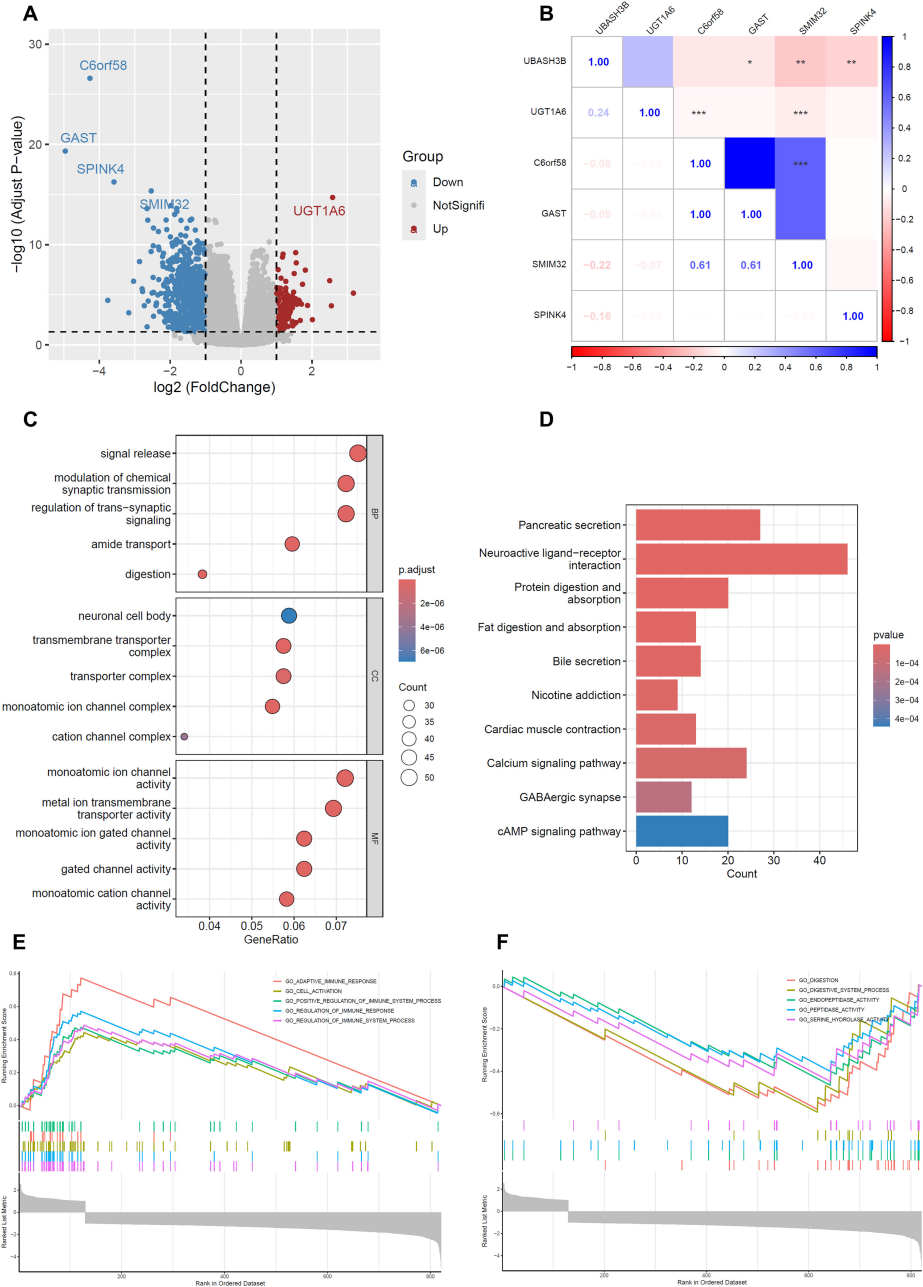
Differential genes and functional enrichment analysis of UBASH3B. Based on TCGA-PAAD cohort data. **(A)** Volcano plot depicting DEGs between high and low UBASH3B expression groups. **(B)** Heatmap showing the correlation between UBASH3B and the expression of C6orf58, GAST, SPINK4, SMIM32, and UGT1A6 genes; *p < 0.05, **p < 0.01, ***p < 0.001 compared to Low_risk group. **(C)** GO enrichment analysis for BP, MF, and CC of DEGs. **(D)** KEGG pathway enrichment analysis of DEGs. **(E, F)** GSEA of DEGs, displaying only the top 5 positively regulated and top 5 negatively regulated pathways.

The analysis identified a total of 838 DEGs, with 709 genes being downregulated and 129 genes upregulated (**Figure 6A**). Based on adjusted p-values, we ranked these genes and highlighted the top five: C6orf58, GAST, SPINK4, SMIM32, and UGT1A6. Notably, except for the upregulated UGT1A6, the other four genes (C6orf58, GAST, SPINK4, and SMIM32) were all downregulated. Further correlation analysis revealed that SMIM32 exhibited the highest expression correlation with UBASH3B, with a correlation coefficient of -0.22 (*p* < 0.001) (**Figure 6B**), indicating that SMIM32 may play a significant role in UBASH3B-mediated biological processes, particularly within the context of PC.

Functional enrichment analysis was performed on the DEGs using GO, KEGG, and GSEA methods to uncover underlying biological processes, pathways, and molecular functions. GO analysis showed that biological processes primarily involved significant changes in signal transduction and synaptic transmission, especially in the regulation of signal release and synaptic transmission. Cellular components were mainly concentrated in pathways related to neuronal cell bodies and ion channels. Molecular functions were enriched in ion channel and transport-related pathways (**Figure 6C**). KEGG analysis revealed several signaling pathways closely associated with PC, including pancreatic secretion, neuroactive ligand-receptor interaction, protein and fat digestion and absorption, bile secretion, nicotine addiction, cardiac muscle contraction, calcium signaling pathway, GABAergic synapses, and cAMP signaling pathway (**Figure 6D**). These findings suggest that UBASH3B may be involved in regulating these critical processes. GSEA enrichment analysis further demonstrated the five most significantly upregulated and downregulated pathways. Upregulated pathways predominantly involved multiple aspects of the immune system, such as regulation of immune system processes, immune responses, cell activation, adaptive immune responses, and positive regulation of the immune system (**Figure 6E**), suggesting that UBASH3B may influence the immune status within the tumor microenvironment. Downregulated pathways were concentrated in digestive system and protease activity, including digestive processes, endopeptidase activity, peptidase activity, digestive system processes, and serine hydrolase activity (**Figure 6F**), indicating that changes in UBASH3B expression may interfere with normal digestive system function and protein metabolism.

### Potential role of UBASH3B in the negative regulation of NK cells

**Figure 7:**
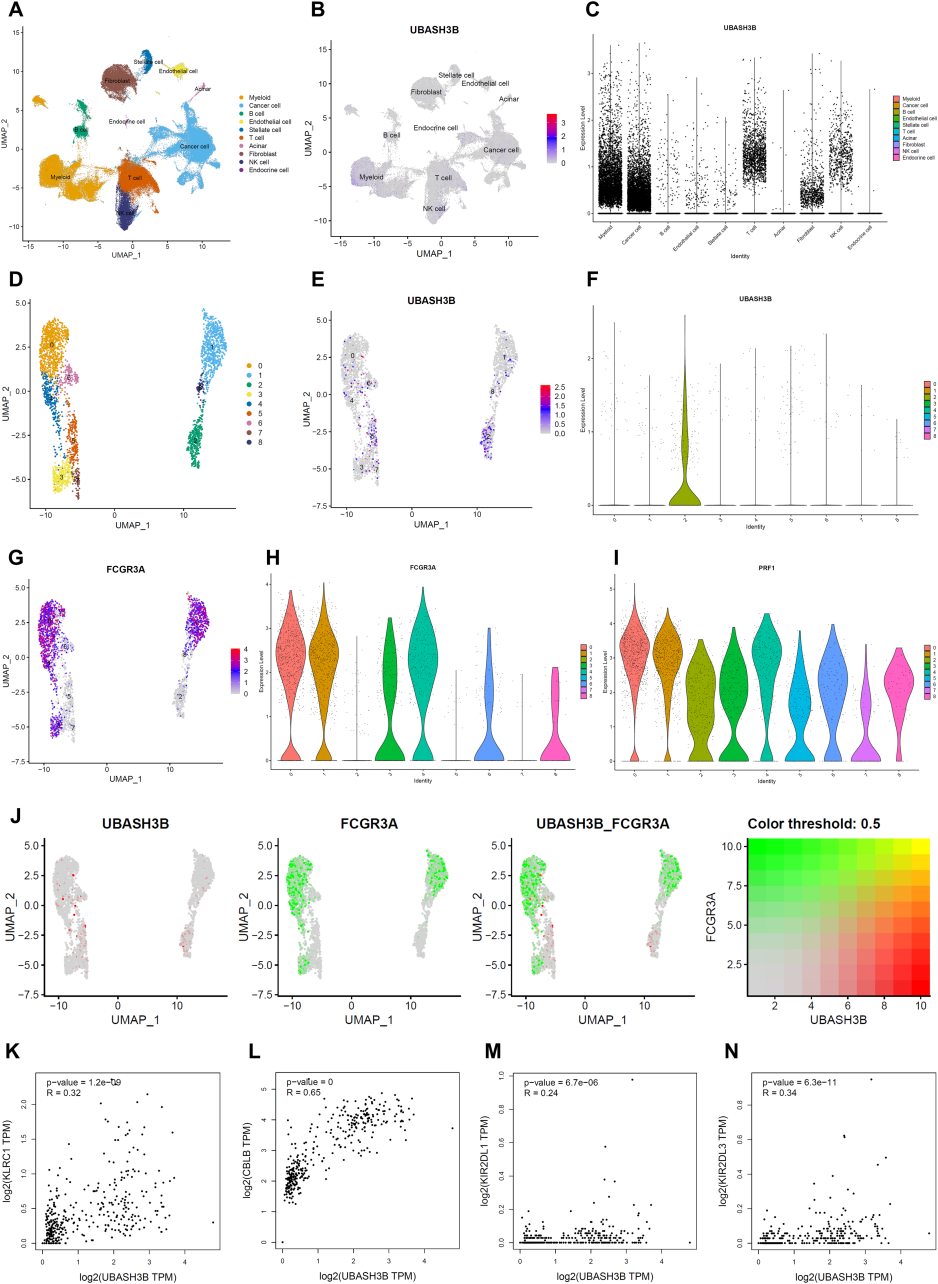
Expression analysis of UBASH3B in cellular subpopulations based on scRNA-seq. **(A)** UMAP clustering plot integrating single-cell RNA sequencing (scRNA-seq) data from 61 pancreatic cancer samples. **(B)** UMAP plot visualizing the expression of the UBASH3B gene across pancreatic cancer cells. **(C)** Violin plots depicting UBASH3B expression levels in various cellular subpopulations within pancreatic cancer. **(D)** UMAP clustering plot for NK cell scRNA-seq data. **(E)** UMAP plot highlighting UBASH3B expression in NK cells. **(F)** Violin plots illustrating UBASH3B expression across different NK cell subpopulations. **(G)** UMAP plot visualizing FCGR3A expression in NK cells. **(H)** Violin plots showing FCGR3A expression levels across NK cell subpopulations. **(I)** Violin plots depicting PRF1 expression levels across NK cell subpopulations. **(J)** Dual feature expression plot comparing UBASH3B and FCGR3A expression in NK cells. **(K-N)** Scatter plots from the GEPIA database demonstrating the correlation between UBASH3B and inhibitory regulatory factors in NK cells, including KLRC1, CBLB, KIR2DL1, and KIR2DL3.

To further investigate the potential role of UBASH3B as an oncogene in PC, this study integrated single-cell sequencing data from 61 PC patients available in the GEO database and conducted a comprehensive analysis. Using known cell subpopulation marker genes, we annotated cells within these samples and identified ten major cell subpopulations: myeloid cells, cancer cells, B cells, endothelial cells, stellate cells, T cells, acinar cells, fibroblasts, NK cells, and endocrine cells (**Figure 7A**; **Supplementary Figure S6A**).

Visualization using FeaturePlot and VlnPlot revealed that UBASH3B was significantly expressed in a subset of NK cells, T cells, myeloid cells, and cancer cells (**Figures 7B, C**). Given previous studies demonstrating UBASH3B’s inhibitory effects on T cell and osteoclast activation (16, 17), and its enrichment in immune-related pathways, we focused our subsequent research on NK cells.

Further analysis revealed that in the re-extracted, dimensionally reduced, and re-clustered NK cells showed that UBASH3B expression was primarily concentrated in cluster 6 and to a lesser extent in cluster 7 (**Supplementary Figures S6B–D**). By analyzing markers such as CD3D, NCAM1, and FCGR3A, we confirmed that clusters 3, 5, 6, and 8 were genuine NK cells, while other clusters were predominantly mixed with T cells (**Supplementary Figures S6E–J**). Subsequently, we performed dimensionality reduction and reclustering on these four clusters, ultimately dividing them into nine new clusters (**Figure 7D**), where UBASH3B was highly expressed in cluster 2 and slightly expressed in cluster 5 (**Figures 7E, F**).

Notably, the NK cell cytotoxicity marker FCGR3A was highly expressed in clusters 0, 1, 3, 4, 6, and 8 but almost absent in clusters 2, 5, and 7 (**Figures 7G, H**), contrasting sharply with the expression pattern of UBASH3B (**Figure 7J**). Similarly, PRF1, a key effector molecule involved in NK cell killing function, also exhibited low expression in clusters 2 and 5 (**Figure 7I**).

Additionally, bulk RNA-seq data analysis revealed a significant positive correlation between UBASH3B and NK cell inhibitory regulatory factors such as KLRC1, CBLB, KIR2DL1, and KIR2DL3 (*p* < 0.001) (**Figures 7K, N**). These findings suggest that UBASH3B may play a role in inhibiting NK cell activity and cytotoxicity, particularly in specific subsets of NK cells.

In summary, our comprehensive analysis provides evidence that UBASH3B is involved in the negative regulation of NK cell function in PC. The distinct expression patterns observed in specific NK cell clusters and the correlations with inhibitory regulatory factors highlight UBASH3B’s potential role in modulating the immune response within the tumor microenvironment. This insight underscores the importance of UBASH3B as a potential therapeutic target for enhancing NK cell-mediated anti-tumor immunity.

### Drug sensitivity analysis of the UBASH3B gene

To investigate the relationship between UBASH3B expression levels and sensitivity to commonly used therapeutic drugs in PC, we analyzed the differences in IC50 values for eight clinically relevant drugs between high- and low-expression groups of UBASH3B. The eight drugs examined were: 5-Fluorouracil-1073, Erlotinib-1168, Gemcitabine-1190, Irinotecan-1088, Oxaliplatin-1089, Oxaliplatin-1806, Paclitaxel-1080, and Trametinib-1372.

**Figure 8:**
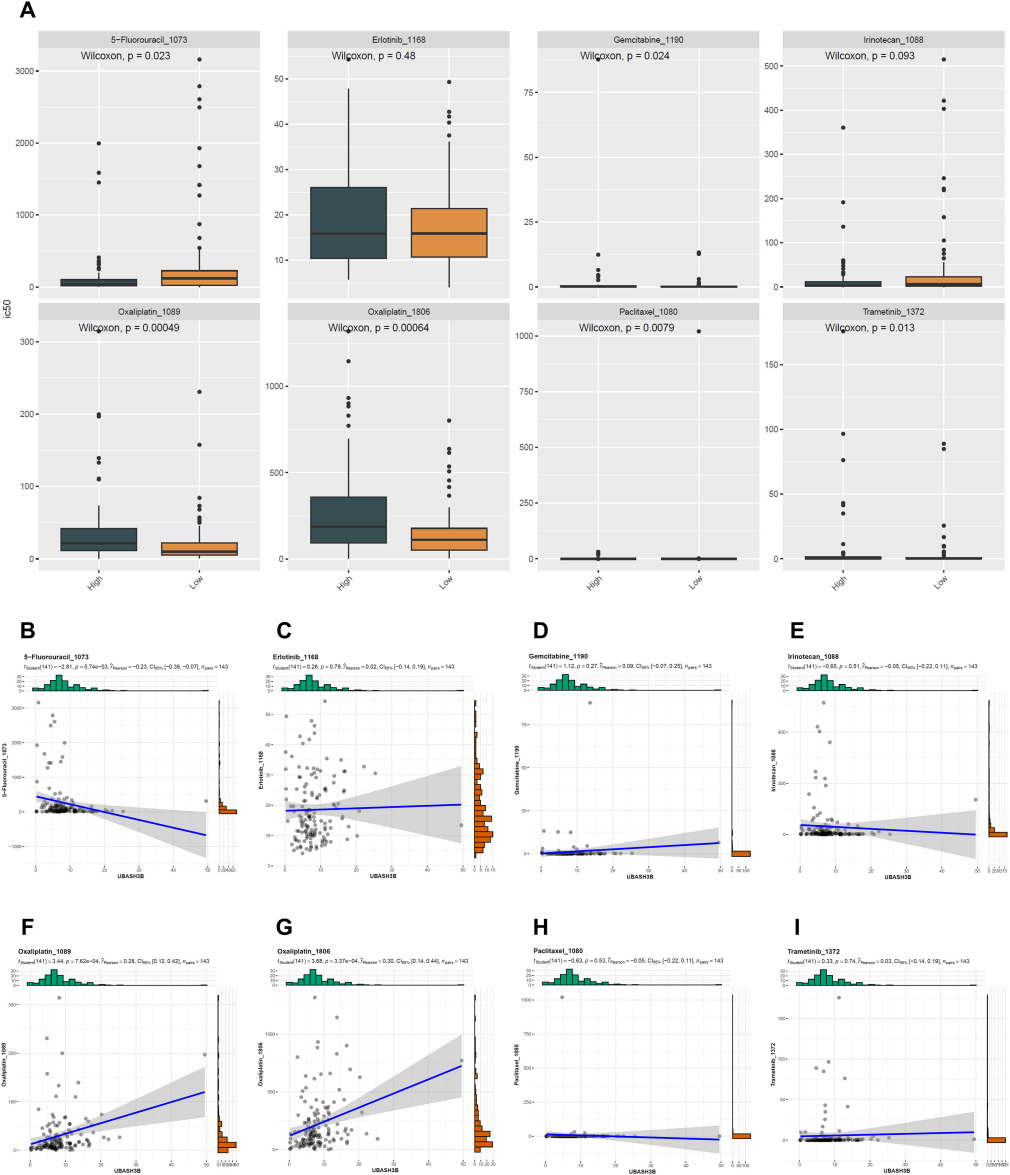
Drug sensitivity analysis based on UBASH3B expression levels. **(A)** Box plots comparing the predicted IC50 values for eight chemotherapeutic drugs between high and low UBASH3B expression groups. The drugs include 5-Fluorouracil-1073, Erlotinib-1168, Gemcitabine-1190, Irinotecan-1088, Oxaliplatin-1089, Oxaliplatin-1806, Paclitaxel-1080, and Trametinib-1372 **(B-I)** Scatter plots showing the correlation between UBASH3B expression levels and the predicted IC50 values for each drug, specifically: **(B)** 5-Fluorouracil-1073, **(C)** Erlotinib-1168, **(D)** Gemcitabine-1190, **(E)** Irinotecan-1088, **(F)** Oxaliplatin-1089, **(G)** Oxaliplatin-1806, **(H)** Paclitaxel-1080, **(I)** Trametinib-1372.

The results showed significant differences in IC50 values between the UBASH3B high- and low-expression groups for six of these drugs (*p* < 0.05), with the exceptions being Erlotinib and Irinotecan (**Figure 8A**). Specifically, the low-expression group exhibited higher sensitivity to five of the drugs but was relatively insensitive to 5-Fluorouracil. Further correlation analysis revealed relationships between UBASH3B expression levels and specific drug IC50 values. UBASH3B expression was positively correlated with the IC50 values of Oxaliplatin (1089 and 1806) (*p* < 0.001) (**Figures 8F, G**) and negatively correlated with the IC50 value of 5-Fluorouracil (*p* < 0.001) (**Figure 8B**). No significant correlations were observed between UBASH3B expression and IC50 values for the other drugs (**Figures 8C–E, H, I**).

These findings suggest that high expression of UBASH3B may be associated with increased drug resistance in PC patients. In clinical decision-making, for patients with high UBASH3B expression, treatment with 5-Fluorouracil might be prioritized; whereas for those with low UBASH3B expression, drugs like Oxaliplatin could be more effective options.

## Discussion

This study delves into the role of innate immune cell barrier-related genes in PC prognosis, particularly through systematic differential expression analysis, enrichment analysis, and machine learning model construction. These analyses reveal the significant potential of innate immune cell barrier genes in predicting PC patient outcomes. Notably, UBASH3B emerges as a novel prognostic marker for PC, playing a critical role in negatively regulating NK cells and correlating with drug sensitivity. Importantly, while bulk RNA-seq analysis may obscure cell-type-specific contributions, our integrative approach—combining deconvolution (CIBERSORT), single-cell validation, and survival modeling—ensured that UBASH3B’s prognostic value reflects intrinsic tumor biology and functionally critical immune subsets rather than confounding cellular heterogeneity.

The innate immune system serves as the host’s first line of defense, comprising physical and chemical barriers along with various immune cells equipped with pattern recognition receptors. These components work together to nonspecifically eliminate abnormal cells or activate adaptive immune responses, thereby delaying tumor development. The cellular barrier primarily includes monocytes/macrophages, neutrophils, NK cells, and dendritic cells, which play a dominant role in innate immunity (4). Ideally, all tumor cells would be cleared by the host immune system; however, tumor cells can evade immune destruction by reducing immunogenicity or releasing immunosuppressive factors (31). Therefore, targeting the tumor immune system appears promising for cancer therapy. Immunotherapies like checkpoint inhibitors and adoptive cell therapies have been widely applied in clinical settings, including anti-PD-1/PD-L1 (32), anti-CTLA-4 (33), and CAR-T therapies (33) targeting the adaptive immune system. However, these treatments have not met expectations for most solid tumors, especially PC (34, 35). Consequently, targeting the innate immune system in PC has gained attention, with studies showing that innate immune-related cells dominate the PC microenvironment (5). While targeting cells like neutrophils (36) and tumor-associated macrophages (37) has shown promise in mouse models, clinical trials have not achieved expected results (8, 9). Thus, a deeper understanding of mechanisms inhibiting innate immune cell function and the roles of related genes in PC is crucial. This study investigated the potential of innate immune cell barrier-related genes as prognostic biomarkers for PC and used machine learning to develop a robust risk prediction model. By integrating data from KEGG, ImmPort Portal, MSigDB, and InnateDB, we identified 1,356 genes associated with the innate immune barrier. Analyzing TCGA and GTEx datasets, which included 178 PC samples and 172 normal pancreatic tissue samples, we identified 3,591 DEGs, intersecting these with the innate immune genes to select those with potential prognostic value.

In PC patients, the relative frequency of NK cells in the blood correlates positively with survival rates, but these cells exhibit lower cytotoxicity compared to healthy individuals (7). This suggests that NK cells play a key role in PC development. Yang et al. further identified a unique subtype of dysfunctional NK cells in PC patients, indicating that prognosis depends not only on NK cell quantity but also on functional subtypes (38). Subsequent GO and KEGG pathway enrichment analyses revealed significant associations between these genes and leukocyte-mediated immune responses, immune effect process regulation, and myeloid leukocyte-specific functions, highlighting the critical role of NK cells in the innate immune barrier of PC. Univariate Cox regression analysis identified 8 protective and 84 risk genes, forming the basis for constructing a robust prognostic model using RSF algorithms. The model demonstrated excellent predictive performance with an average C-index of 0.615 across validation datasets. To validate the clinical utility of our model, we performed univariate and multivariate Cox regression analyses using clinical information from the TCGA cohort. The results demonstrated that the Riskscore serves as a significant independent prognostic factor (*p* < 0.001). Patients were divided into high-risk and low-risk groups based on the median Riskscore value. Kaplan-Meier survival analysis revealed a significant difference in survival status and duration between the two groups, with the low-risk group showing superior overall survival across multiple cohorts (*p* < 0.01). Furthermore, ROC curve analysis indicated excellent performance in predicting 3-year and 5-year survival rates, with AUC values approaching or exceeding 0.9, thus confirming the model’s robust long-term predictive capability.

Nerve recruitment represents a defining characteristic of malignant neoplasms, with perineural infiltration being a particularly prominent feature in PC, where it occurs in approximately 90% of cases (39). This neuroinvasive phenomenon is strongly associated with increased risks of local recurrence and metastatic progression in PC (40). Notably, experimental models have demonstrated that neural invasion actively promotes PC in murine systems (41). Emerging evidence underscores the critical role of neural signaling in tumor biology, as neoplastic-nerve crosstalk can orchestrate tumor progression through modulation of the tumor microenvironment, enhance metastatic potential, and influence chemoradiotherapy responsiveness (42). Among the various neural pathways implicated, GABAergic signaling has emerged as a key regulatory axis in oncogenesis, with documented effects on tumor proliferation, metastasis, stemness maintenance, and microenvironment modulation (43). However, the precise mechanisms governing its role in PC remain poorly understood, representing a critical gap in current research. Functional enrichment analysis based on Riskscore differences showed that high-risk and low-risk groups differed significantly in molecular functions related to ion balance and metabolic regulation, neural signaling, and intercellular communication, particularly the GABAergic synapse pathway. GSEA analysis further revealed biological process differences, such as epithelial tissue development and differentiation being more prominent in the high-risk group, while low-risk groups focused on neuronal and synaptic functions and material transport processes. This suggests that neural signaling plays a crucial role in PC development.

TMB analysis indicated that the high-risk group exhibited significantly higher TMB, reflecting greater genomic instability. Immune infiltration analysis showed lower immune scores and stromal scores in the high-risk group, suggesting reduced immune infiltration. Immune cell composition analysis using the “CIBERSORT” package found that B cells, plasma cells, CD8+T cells, and monocytes were significantly less abundant in the high-risk group, supporting the notion of weakened immune infiltration. Drug sensitivity analysis revealed that the high-risk group had higher IC50 values for Erlotinib-1168, Oxaliplatin-1089, and Oxaliplatin-1806 but lower IC50 values for 5-Fluorouracil-1073, indicating higher resistance overall but increased sensitivity to 5-Fluorouracil. The differential drug sensitivity patterns, particularly the increased 5-Fluorouracil responsiveness in high-risk patients, carry direct therapeutic implications. This subgroup, characterized by innate immune dysfunction and metabolic reprogramming, may benefit from preferential use of 5-Fluorouracil-based regimens to exploit both direct cytotoxic effects and indirect immune-potentiating mechanisms. Notably, the standard FOLFIRINOX regimen already includes 5-Fluorouracil, suggesting our risk model could optimize patient selection for this intensive therapy. However, no current studies stratifying PC patients using innate immune biomarkers for chemotherapy allocation - a critical gap our findings aim to address. Future trials could leverage this risk model to design biomarker-driven protocols comparing conventional vs. risk-adapted 5-Fluorouracil dosing schedules.

In 2004, Nick Carpino et al. (44) first identified UBASH3B as a negative regulator of T-cell receptors. Subsequent research confirmed its protein tyrosine phosphatase activity and demonstrated that it modulates T-cell activity by inhibiting proximal T-cell receptor signaling. This discovery laid the foundation for understanding UBASH3B’s role in immune regulation. Further studies revealed that UBASH3B’s function extends beyond T cells; it also exerts negative regulatory effects in macrophages (45). Additionally, UBASH3B has been detected in dendritic cells and mast cells (46, 47), suggesting its involvement in broader immune response mechanisms. Despite these important findings, the specific functions and molecular mechanisms of UBASH3B remain incompletely understood, particularly its role in NK cells, which remains underexplored. Clinically, current research on UBASH3B primarily focuses on autoimmune diseases and chronic inflammation, highlighting its potential importance in immune-related disorders. In contrast, studies on UBASH3B’s role in cancer, especially PC, are relatively scarce (12, 48, 49), with no systematic reports available.

In this study, we screened key genes from the best-performing RSF model and identified ITGB6, COL17A1, MMP28, DIAPH3, and UBASH3B as the top five critical genes. Analysis using the GEPIA database and multivariate Cox regression revealed that these genes play significant roles in PC development and are closely associated with patient overall survival. These genes are mechanistically linked to hallmark pathological processes of PC, as supported by literature and pathway analyses. For instance, ITGB6 drives malignant behaviors in PC, with *in vitro* and *in vivo* studies demonstrating that ITGB6 knockdown suppresses proliferation, invasion, and migration of pancreatic cancer cells by disrupting TGF-β signaling and epithelial-mesenchymal transition (EMT) (27). Similarly, COL17A1 promotes tumor aggressiveness, as COL17A1 depletion inhibits proliferation, migration, and invasion through modulation of extracellular matrix (ECM) remodeling and chemoresistance pathways (28). MMP28 facilitates tumor progression via TGF-α/EGFR axis activation, where elevated MMP28 expression enhances TGF-α maturation to drive oncogenic signaling and metastasis (50). DIAPH3 sustains redox homeostasis in PC by upregulating thioredoxin reductase 1 (TrxR1), which reduces cellular reactive oxygen species (ROS) levels to maintain malignant phenotypes and chemoresistance (30). Notably, our study is the first to demonstrate the correlation between UBASH3B expression and PC prognosis. We found that UBASH3B expression is significantly related to NK cell infiltration, particularly showing a negative correlation with NK cell activation. This suggests that UBASH3B may act as a negative regulator in NK cell-mediated immune responses, deepening our understanding of the PC immune microenvironment and providing valuable insights for future therapeutic targets.

To further explore the potential mechanisms underlying UBASH3B’s role as a poor prognostic factor in PC, we conducted differential expression analysis and functional enrichment analysis using RNA-seq data from the TCGA-PAAD database. Our results indicate that changes in UBASH3B expression may interfere with normal digestive system function and protein metabolism, impacting the immune state within the tumor microenvironment. Comprehensive analysis revealed that UBASH3B likely inhibits cell activity and cytotoxicity in specific NK cell subsets, notably those highly expressed in cluster 2. Studies have demonstrated that blocking the KLRC1/HLA-E pathway in PC significantly enhances the anti-tumor activity of effector cells, thereby suppressing tumor progression (51). KIR2DL1 and KIR2DL3, receptors expressed on NK cells, primarily recognize HLA-C molecules to inhibit NK cell cytotoxicity. Their high expression in anti-tumor immunity suppresses the cytotoxic function of NK cells (52). CBLB, an E3 ubiquitin ligase, mediates protein ubiquitination to inhibit the activation of T cells and NK cells (53). In this study, UBASH3B exhibited significant positive correlations with KLRC1, CBLB, KIR2DL1, and KIR2DL3 (p < 0.001), suggesting its potential role in suppressing NK cell cytotoxicity.

Moreover, UBASH3B expression levels correlate significantly with sensitivity to multiple commonly used therapies, suggesting its potential value in personalized treatment strategies for PC. UBASH3B distinguishes itself from canonical PC biomarkers through its unique position at the intersection of innate immune dysfunction and therapeutic vulnerability. Unlike KRAS mutations that reflect tumor-intrinsic signaling or CA19–9 as a nonspecific secretory product, UBASH3B expression directly quantifies NK cell-mediated immunosuppression while predicting differential chemotherapy responses. This dual functionality enables risk stratification beyond conventional TNM staging and informs therapeutic selection. Crucially, UBASH3B’s association with conserved immune checkpoint pathways (**Figure 7**) positions it as a biomarker for emerging NK cell therapies.

In summary, this study provides new theoretical foundations and technical support for precision medicine in PC, highlighting the critical roles of innate immune cell barrier-related genes, especially UBASH3B, in prognosis and treatment. Through systematic analyses and machine learning, we identified key genes impacting patient outcomes and developed a robust prognostic risk model. While bulk RNA-seq has inherent limitations in resolving cellular heterogeneity, our multi-modal approach—integrating deconvolution, single-cell analysis, and drug sensitivity profiling—ensured robust identification of biologically relevant targets. However, several limitations exist: the sample size may be limited for specific immune cell subtypes, and further mechanistic studies are needed to fully understand the roles of these genes, particularly UBASH3B in NK cell function. Additionally, the clinical utility of our risk prediction model requires validation in prospective studies. To address this question, our team plans to initiate a multicenter observational trial to validate the model through standardized treatment protocols in 300 consecutively enrolled patients with PC. This study will specifically assess: 1) the model’s prognostic accuracy at first diagnosis, and 2) its dynamic predictive value throughout the course of a chemotherapy cycle. Future technological advancements will integrate circulating tumor DNA (ctDNA) profiling and enhanced radiomic analysis of CT scans to develop a multimodal predictive platform. Meanwhile, future work leveraging spatial transcriptomics or NK cell-specific knockout models will clarify UBASH3B’s spatial and functional roles in PC progression. Addressing these limitations will enhance the robustness and clinical relevance of our findings, paving the way for more effective and personalized therapies for PC patients.

## Conclusion

This study delves into the role of innate immune cell barrier-associated genes in predicting patient outcomes, particularly in PC, uncovering their significant potential in forecasting survival. Through systematic differential expression analysis, enrichment analysis, and the construction of machine learning models, we have identified a set of key genes, notably UBASH3B, as novel prognostic biomarkers. Our findings underscore UBASH3B’s critical role in negatively regulating NK cell activity and its association with drug sensitivity.

Our research indicates that innate immune cell barrier genes, especially UBASH3B, may modulate the tumor microenvironment by influencing NK cell function and infiltration, thereby impacting the prognosis of PC patients. By integrating resources from multiple public databases, we developed a robust risk prediction model that exhibits outstanding long-term predictive power. The model confirms that the Riskscore serves as a significant independent prognostic factor. Moreover, its ability to distinctly categorize high-risk and low-risk patients provides a foundation for personalized treatment strategies. Notably, the expression levels of UBASH3B are not only inversely correlated with NK cell activation but also associated with sensitivity to several common therapies, suggesting its potential as a new therapeutic target in PC. Despite limitations such as limited sample sizes for specific immune cell subtypes, this study establishes a theoretical basis and technical support for the advancement of precision medicine in PC, highlighting the importance of innate immune cell barrier-related genes in both prognostic assessment and therapeutic intervention.

Future studies should aim to validate these findings and further explore the specific mechanisms and clinical applications of UBASH3B and other key genes, with the goal of enhancing diagnostic accuracy and treatment efficacy for PC patients. Additionally, prospective studies are crucial for evaluating the clinical utility of our risk prediction model, ultimately striving to provide more effective and personalized medical solutions for PC patients.

## Data Availability

The raw data supporting the conclusions of this article will be made available by the authors, without undue reservation.
