# Supplementary material for: Innate immune cell barrier-related genes inform precision prognosis in pancreatic cancer
Source: Front Immunol. 2025 May 23;16:1559373. doi: 10.3389/fimmu.2025.1559373 (PMC12141296; doi:10.3389/fimmu.2025.1559373)
Supplement: Supplementary file 1 [file DataSheet1.pdf]

Innate Immune Cell Barrier-related Genes Inform Precision Prognosis in Pancreatic Cancer

Qiang Luo[1,2], Tingting Jiang[1,2], Dacheng Xie[3], Xiaojia Li[1,2], Keping Xie[1,2]

[1]Center for Pancreatic Cancer Research, The South China University of Technology School of Medicine, Guangzhou, Guangdong 510006, China

[2]The South China University of Technology Comprehensive Cancer Center, Guangzhou, Guangdong 510006, China

[3]Department of Medical Oncology, Shanghai Pulmonary Hospital & Thoracic Cancer Institute, Tongji University School of Medicine, Shanghai 200433, China

[*]*Correspondence authors*: Keping Xie or Xiaojia Li, Center for Pancreatic Cancer Research, South China University of Technology School of Medicine, Guangzhou 510006, China; *E-mail*: scutmedicine@scut.edu.cn or xiaojia0424@scut.edu.cn.

Disclosure of Potential Conflicts of Interest: The authors declare no conflict of interest.

Financial Support: The work is partly supported by National Natural Science Foundation of China (#82072632) and Guangzhou Municipality Bureau of Science and Technology, Guangzhou, China (#202102010033) and Natural Science Foundation of Guangdong Province, China (#2022A1515012585).

[#]Q. Luo and T. Jiang have contributed equally to this work and are considered co-first authors.

Table of contents

Supplementary Figure S1. Batch effect correction on gene expression data distribution.. 3

Supplementary Figure S2. scRNA-seq data preprocessing and batch effect correction ... 4

Supplementary Figure S3. GO enrichment analysis of Cell-DEG-down and Cell-DEG-up 5

Supplementary Figure S4. Differences in immune infiltration and drug sensitivity between high- and low-risk groups 6

Supplementary Figure S5. Association of ITGB6, COL17A1, MMP28, and DIAPH3 gene expression with pancreatic cancer prognosis 7

Supplementary Figure S6. Expression analysis of UBASH3B in cellular subpopulations based on scRNA-seq 8

Supplementary Table S1. Clinical characteristics of GEO cohorts 10

Supplementary Table S2. Systematic Comparison Between TCGA-PAAD and GEO Validation Cohorts 10

Supplementary Table S3. Algorithm Selection Rationale and Hyperparameter Tuning Strategies for 14 Machine Learning Models 10

Supplementary Table S4. Lists of CDGs, CDPGs, and CDRGs 10

Supplementary Table S5. C-Indices for Training and Validation Sets of 162 Machine Learning Models Constructed Using CDGs, CDPGs, and CDRGs 10

Supplementary Table S6. IC50 Predictions for 198 Drugs in TCGA-PAAD Cohort Using the “oncoPredict” Package 10

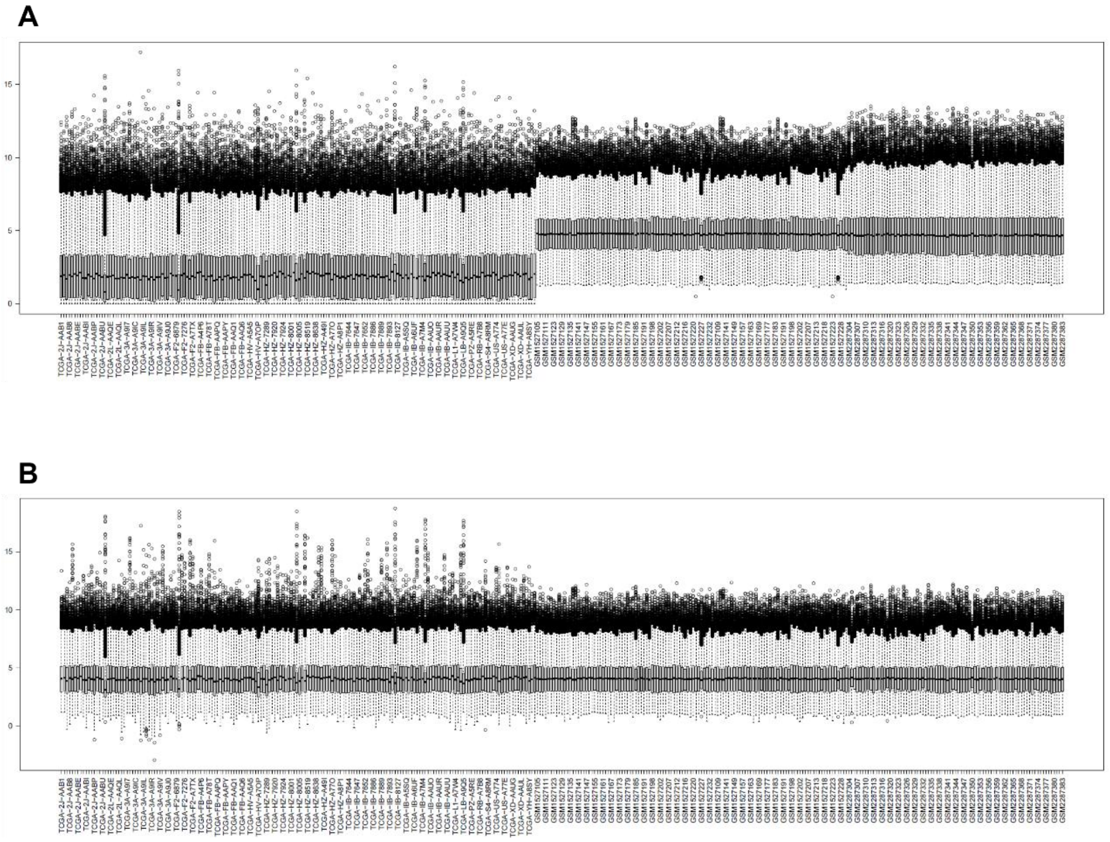

Supplementary Figure S1. Batch effect correction on gene expression data distribution

Each boxplot represents the distribution of gene expression values across samples. Panel (**A**) shows raw data with batch-specific offsets, while panel (**B**) demonstrates the alignment of distributions post-normalization.

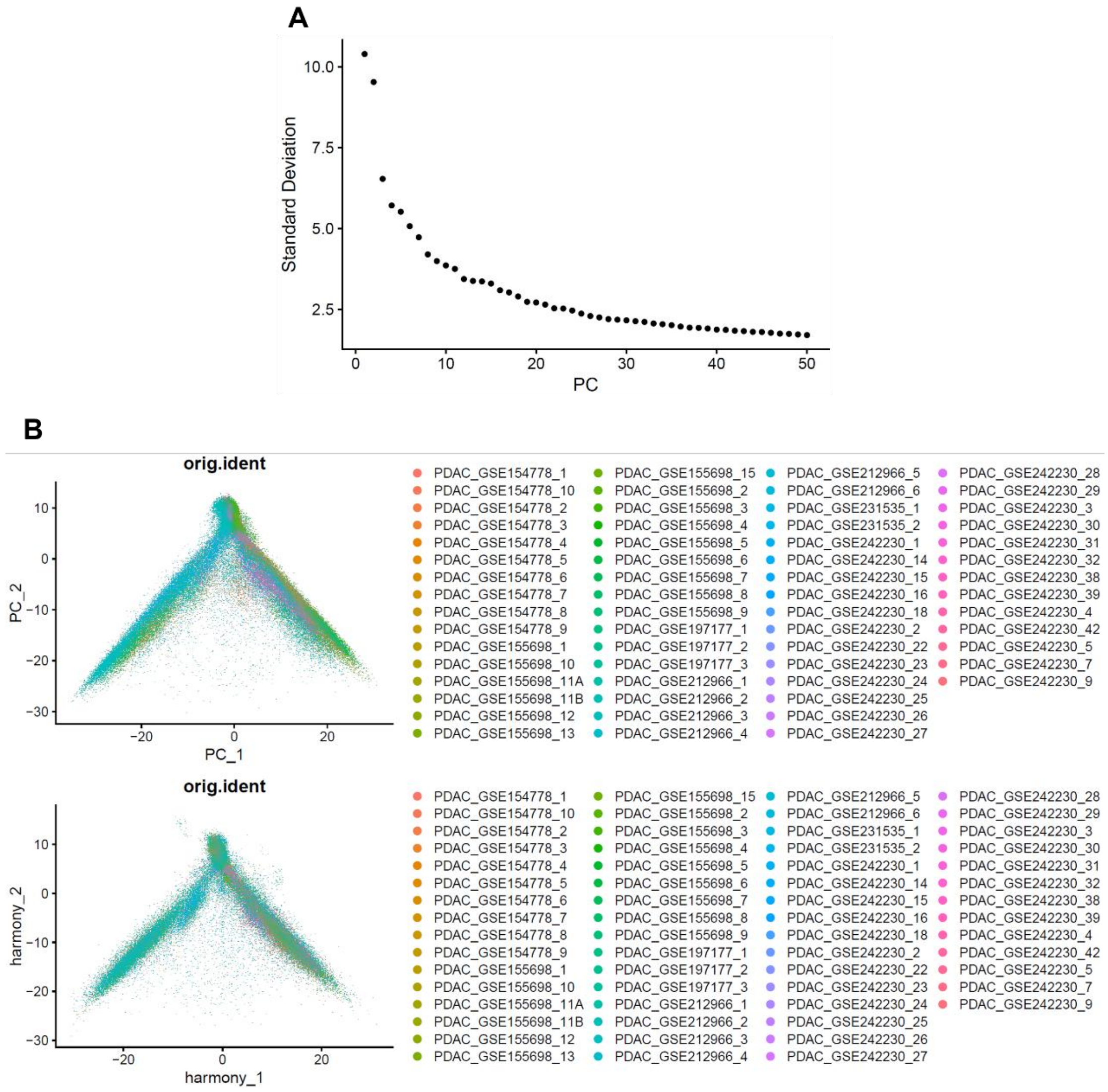

Supplementary Figure S2. scRNA-seq data preprocessing and batch effect correction

(**A**) Principal Component Analysis (PCA) Optimization via Elbow Method. (**B**) Batch Effect Removal and Biological Structure Preservation via Harmony Algorithm.

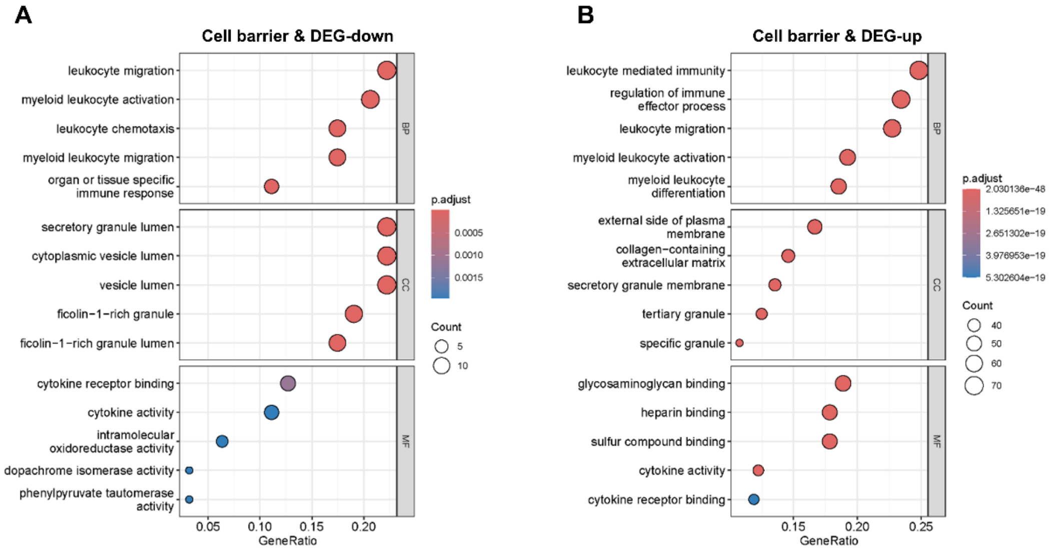

Supplementary Figure S3. GO enrichment analysis of Cell-DEG-down and Cell-DEG-up

(**A**) GO enrichment analysis for downregulated genes (Cell-DEG-Down), showing the top five enriched pathways in each of the three GO categories: Biological Process (BP), Cellular Component (CC), and Molecular Function (MF). (**B**) GO enrichment analysis for upregulated genes (Cell-DEG-Up), displaying the top five enriched pathways in each of the three GO categories: BP, CC, and MF.

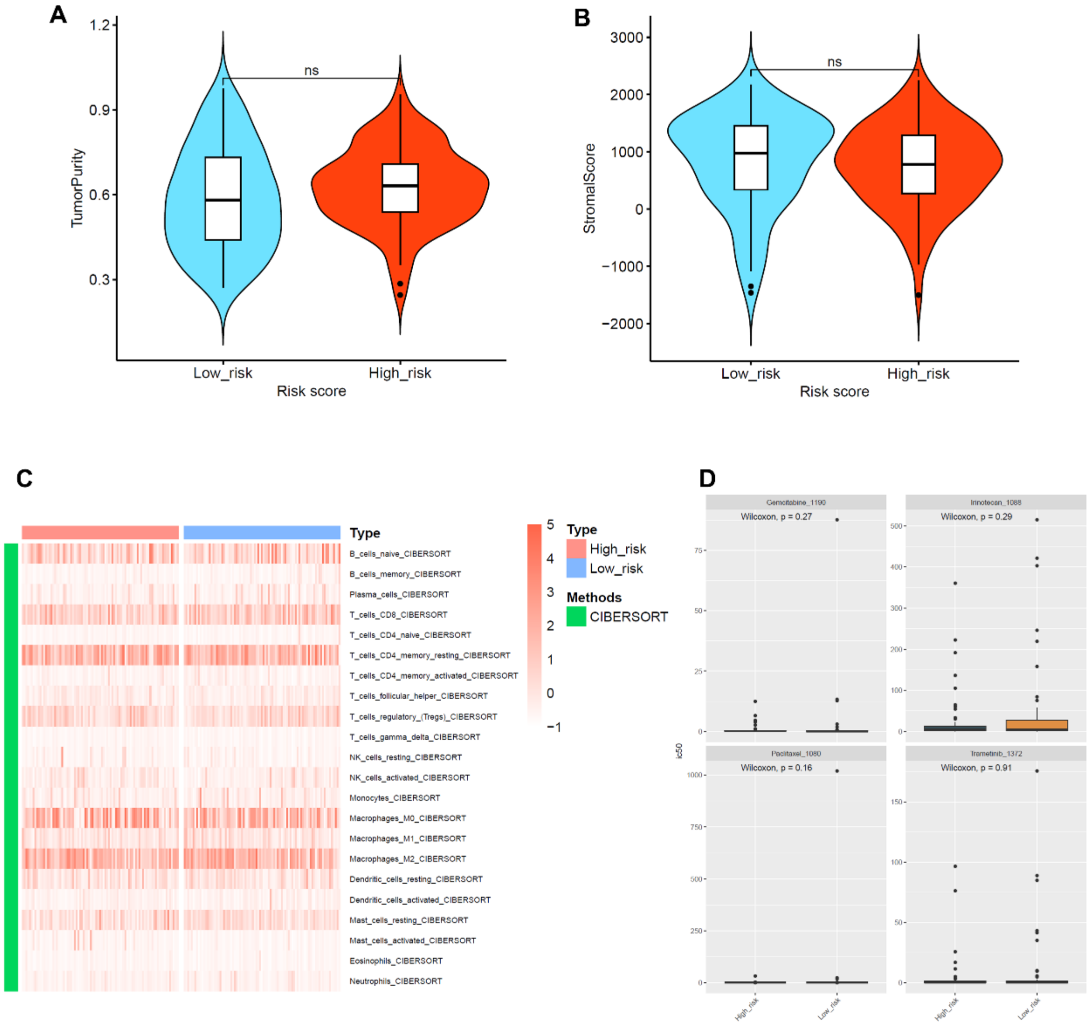

Supplementary Figure S4. Differences in immune infiltration and drug sensitivity between high- and low-risk groups

Based on TCGA-PAAD cohort data. (**A**, **B**) Violin plot showing the distribution of TumorPurity and StromalScore from ESTIMATE analysis between high- and low-risk groups. (**C**) Heatmap displaying the results of CIBERSORT analysis for immune cell infiltration in high- and low-risk groups. (**D**) Box plots comparing the predicted IC50 values for Gemcitabine-1190, Irinotecan-1088, Paclitaxel-1080, and Trametinib-1372 between high- and low-risk groups.

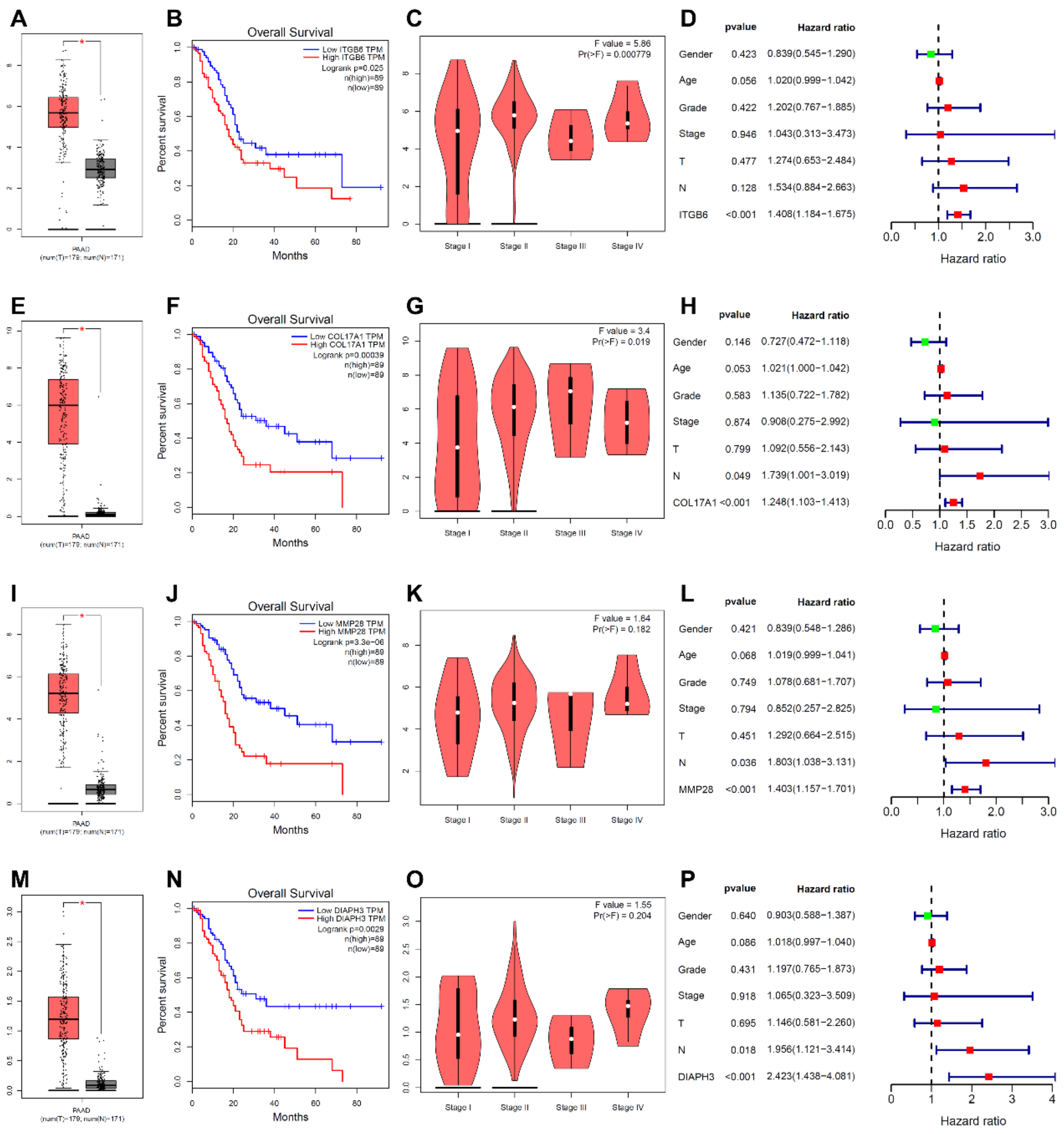

Supplementary Figure S5. Association of ITGB6, COL17A1, MMP28, and DIAPH3 gene expression with pancreatic cancer prognosis

(**A**, **E**, **I**, **M**) Box plot from the GEPIA database depicting ITGB6, COL17A1, MMP28, and DIAPH3 gene expression levels. (**B**, **F**, **J**, **N**) Kaplan-Meier survival curve for ITGB6, COL17A1, MMP28, and DIAPH3 expression from the GEPIA database. (**C**, **G**, **K**, **O**) Violin plot illustrating stage-wise analysis of ITGB6, COL17A1, MMP28, and DIAPH3 expression from the GEPIA database. (**D**, **H**, **L**, **P**) Forest plot from multivariate Cox regression analysis based on ITGB6, COL17A1, MMP28, and DIAPH3 expression data and clinical information from the TCGA-PAAD cohort.

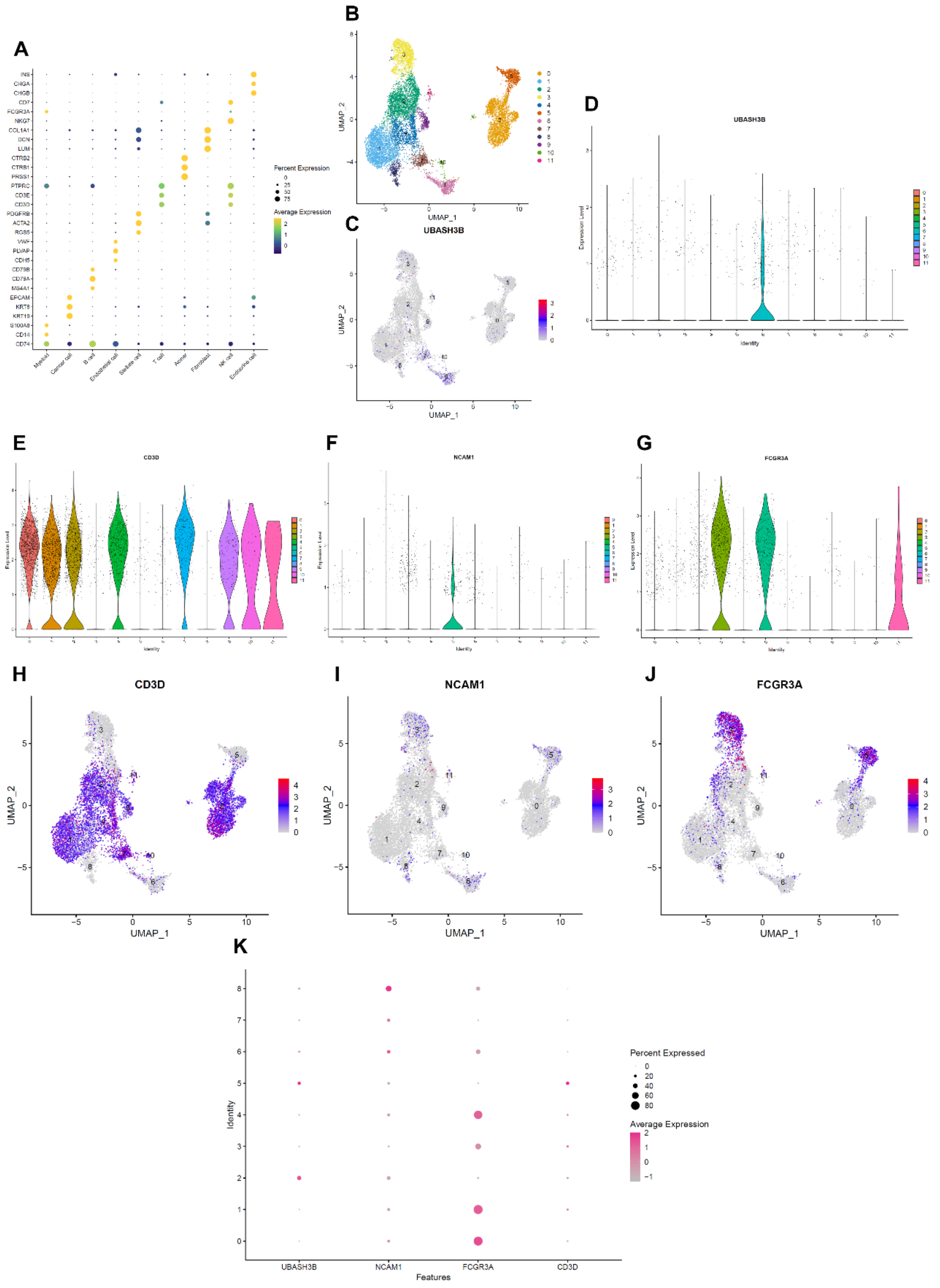

Supplementary Figure S6. Expression analysis of UBASH3B in cellular subpopulations based on scRNA-seq

(**A**) Bubble plot showing the expression of marker genes in clusters derived from pancreatic cancer scRNA-seq data. (**B**) UMAP clustering plot for NK cell scRNA-seq data, illustrating distinct cellular subpopulations. (**C**) UMAP plot highlighting UBASH3B

expression levels across NK cells. (**D**-**G**) Violin plots depicting the expression of UBASH3B, CD3D, NCAM1, and FCGR3A across different NK cell subpopulations. (**H**-**J**) UMAP plots highlighting the expression of CD3D, NCAM1, and FCGR3A within NK cells. (**K**) Bubble plot showcasing the expression of UBASH3B, CD3D, NCAM1, and FCGR3A across various NK cell subpopulations.

Supplementary Table S1. Clinical characteristics of GEO cohorts

Contains cohorts GSE62452, GSE78229, and GSE85916.

Supplementary Table S2. Systematic Comparison Between TCGA-PAAD and GEO Validation Cohorts

This table provides a systematic comparison of key characteristics between the TCGA-PAAD dataset and three GEO validation cohorts (GSE62452, GSE782329, GSE85916). The comparison includes the following dimensions: Sample Size; Technical Platform, Tissue Type, Clinical Characteristics, Survival Data, Geographic Distribution.

Supplementary Table S3. Algorithm Selection Rationale and Hyperparameter Tuning Strategies for 14 Machine Learning Models

This table provides two sections detailing the rationale for algorithm selection and hyperparameter tuning strategies. Sheet 1: Algorithm Selection Rationale; Sheet 2: Hyperparameter Tuning.

Supplementary Table S4. Lists of CDGs, CDPGs, and CDRGs

Supplementary Table S5. C-Indices for Training and Validation Sets of 162 Machine Learning Models Constructed Using CDGs, CDPGs, and CDRGs

This table summarizes the c-indices for 162 machine learning models trained using candidate differentially expressed genes (CDGs), protective prognostic genes (CDPGs), and risk genes (CDRGs). The c-index measures the predictive accuracy of each model in both training and validation sets.

Supplementary Table S6. IC50 Predictions for 198 Drugs in TCGA-PAAD Cohort Using the “oncoPredict” Package

Sheet1: Contains IC50 predictions for 198 drugs across all samples in the TCGA-PAAD cohort. Columns are named after drug names, rows are named after sample IDs, and a “sample_group” column indicates group assignment based on RiskScore.

Sheet2: Summarizes the average IC50 prediction values for low-risk and high-risk groups for each drug. The p-values were calculated using the Wilcoxon rank-sum test to compare the IC50 values between the two groups.
